# Socioeconomic and Behavioral Correlates of COVID-19 Infections among Hospital Workers in the Greater Jakarta Area, Indonesia: A Cross-Sectional Study

**DOI:** 10.3390/ijerph18105048

**Published:** 2021-05-11

**Authors:** Adrianna Bella, Mochamad Thoriq Akbar, Gita Kusnadi, Olivia Herlinda, Putri Aprilia Regita, Dian Kusuma

**Affiliations:** 1 Center for Indonesia’s Strategic Development Initiatives (CISDI), Jakarta 10350, Indonesia; adrianna.bella@cisdi.org (A.B.); thoriqakbarr@gmail.com (M.T.A.); gita.kusnadi@cisdi.org (G.K.); olivia.herlinda@cisdi.org (O.H.); putriregita@cisdi.org (P.A.R.); 2 Centre for Health Economics & Policy Innovation, Imperial College Business School, London SW7 2AZ, UK

**Keywords:** socioeconomic, protective behaviors, COVID-19, healthcare workers, hospital, Indonesia

## Abstract

(1) Background: because of close contacts with COVID-19 patients, hospital workers are among the highest risk groups for infection. This study examined the socioeconomic and behavioral correlates of COVID-19 infection among hospital workers in Indonesia, the country hardest-hit by the disease in the Southeast Asia region. (2) Methods: we conducted a cross-sectional study, which collected data from 1397 hospital staff from eight hospitals in the Greater Jakarta area during April–July 2020. The data was collected using an online self-administered questionnaire and Reverse Transcription-Polymerase Chain Reaction (RT-PCR) tests. We employed descriptive statistics and adjusted and unadjusted logistic regressions to analyze the data of hospital workers as well as the subgroups of healthcare and non-healthcare workers. (3) Results: from a total of 1397 hospital staff in the study, 22 (1.6%) were infected. In terms of correlates, being a healthcare worker (adjusted odds ratio (AOR) = 8.31, 95% CI 1.27–54.54) and having a household size of more than five (AOR = 4.09, 1.02–16.43) were significantly associated with a higher risk of infection. On the other hand, those with middle- and upper-expenditure levels were shown to have a lower risk of infection (AOR = 0.06, 0.01–0.66). Behavioral factors associated with COVID-19 infection among healthcare and non-healthcare workers included knowledge of standard personal protective equipment (PPE) (AOR = 0.08, 0.01–0.54) and application of the six-step handwashing technique (AOR = 0.32, 0.12–0.83). (4) Conclusion: among hospital staff, correlates of COVID-19 infection included being a healthcare worker, household size, expenditure level, knowledge and use of PPE, and application of appropriate hand washing techniques.

## 1. Introduction

Since being officially declared as a global pandemic by the World Health Organization (WHO) in March 2020, coronavirus disease 2019 (COVID-19) has infected over 128.5 million people and has caused more than 2.8 million deaths in 206 countries worldwide by 31 March 2021 [1]. With the burden of the currently existing public health issue, the consequences of this pandemic have been well predicted to be suffered the most by the developing countries compared to their developed counterparts [2]. Despite the implementation of activity restrictions as well as individual and communal protective behaviors at the national and regional levels [3,4], Indonesia has become the country worst-hit by COVID-19 by having the highest number of cases in the South East Asia region in addition to being among the highest mortality rates in the world [5]. As of 31 March 2021, the government has reported over 1.5 million confirmed cases of COVID-19 with 40,858 deaths since the first case was detected on 2 March 2020 [1].

Because of the close contact with COVID-19 patients, those working in healthcare facilities, both healthcare and non-healthcare staff, are among the highest risk groups for infection by COVID-19 [6]. Some studies have found that workers in health facilities have a higher risk of COVID-19 infection than the general population [7,8]. Globally, there were 152,888 healthcare workers recorded as being infected by 8 May 2020 [9]. In Indonesia, a report by the Medical Association revealed that 654 healthcare workers died because of COVID-19 by January 2021 [10]. This has put Indonesia in first and third place in the region and in the world, respectively, in terms of the COVID-19 fatality rate among healthcare workers [11]. With the low healthcare-workers–population ratio, it has been estimated that the country’s healthcare workers have an increased risk of the virus because of high exposure [12]. Considering their critical role in the front line, it is important to understand the correlates of morbidity and mortality among healthcare workers and non-healthcare workers in health facilities in Indonesia.

Previous studies have explored several risk factors related to the previous and current coronavirus infection among hospital staff and/or healthcare workers. Looking back to the SARS-CoV 1 and MERS CoV epidemics, close contact with infected patients, use of PPE, and infection control training turned out to be the predominant risk factors for virus transmission among hospital staff [13,14,15]. In line with the previous epidemics, close contact with infected patients, working in emergency units, overworking, older age, having poor personal protective equipment (PPE), training guidance provision from hospitals, and poor hand hygiene have been found as correlates of COVID-19 infection among healthcare workers [8,9,16,17,18,19,20,21,22]. However, most of these studies were conducted in high-income countries [8,9,17,18,19]. Studies examining the determinants of COVID-19 infections among healthcare workers in low- and middle-income countries (LMICs) were only conducted in China [16,20,21,22]. Thus, our study aims to fill the gap by examining demographic and behavioral correlates of COVID-19 infections among hospital workers in Indonesia, an upper-middle-income country. We hypothesized that COVID-19-related protective behaviors may lower the infection risks, while demographic characteristics may have various significances and relationship directions.

## 2. Methods

### 2.1. Study Design and Data

**Figure 1:**
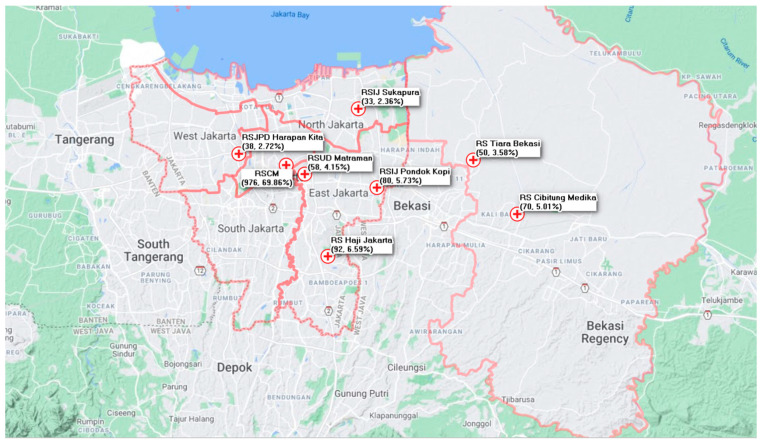
Participating hospitals in the Greater Jakarta Area.

This was a cross-sectional study involving 1397 participants, which included healthcare and non-healthcare hospital workers in eight hospitals in the Greater Jakarta Area (Figure 1), the capital of Indonesia. The area was chosen for several considerations: (1) Jakarta has been one of the epicenters of COVID-19 transmission in Indonesia, which has had relatively high COVID-19 cases since the beginning of the pandemic [1], and (2) as a metropolitan city, Jakarta contains many risk factors for COVID-19 infection, such as poor air pollution [23,24] and severe overcrowding [25].

The primary data collection was conducted from 9 April–1 July 2020. The participants were selected through two channels: partnership agreement and online recruitment. Participants from five hospitals were recruited through a partnership agreement with the Center for Indonesia’s Strategic Development Initiatives (CISDI), whereas the rest were recruited online. An online recruitment was posted on social media to attract hospitals interested in getting free Reverse Transcription-Polymerase Chain Reaction (RT-PCR) tests for their staff. The inclusion criteria for the hospitals included being a COVID-19 referral hospital, having staff with confirmed COVID-19 cases for the past 14 days, and not receiving any access to regular RT-PCR tests from the government. In the recruitment process of participants in each hospital, we suggested including healthcare and non-healthcare workers with the following criteria: (1) had close contact with at least one COVID-19 patient and (2) developed COVID-19 related symptoms. However, in practice, we had minimum control to select the participants based on those criteria.

Participants were asked to fill out a self-administered questionnaire to collect information regarding demographic characteristics and protective behaviors. The data on SARS-CoV-2 infection were obtained based on oropharyngeal and nasopharyngeal swab specimens by trained healthcare workers at participating hospitals. All specimens were sent for RT-PCR testing to the University of Indonesia Clinical Microbiology Lab, which is among the first few laboratories appointed as a COVID-19 laboratory in Indonesia. Results of the self-assessed questionnaire and the tests were matched and analyzed.

### 2.2. Study Variables

The primary dependent variable was COVID-19 infection (1 = positive, 0 = otherwise). An additional dependent variable was having at least one of the main COVID-19 symptoms. The UK National Health Service recommends anyone who experiences one of these main symptoms to get an immediate COVID-19 test: a high temperature, a continuous cough, partial/complete loss of the sense of smell, or partial/complete loss of the sense of taste [26].

The independent variables included two groups: sociodemographic characteristics and protective behaviors. First, sociodemographic variables included sex, being a healthcare worker, age, household size, expenditure level, and smoking status. Under National Law 36/2014, healthcare workers include doctors, dentists, nurses, pharmacists, laboratory staff, and medical interns/residents. The age groups included young adults (19–24 years of age), adults (25–44 years of age), and those middle-aged and over (>44 years of age). The expenditure (expenditure was used as a proxy of income since the data of self-stated income tends to be undervalued) levels included poor, vulnerable, aspiring middle class, middle class, and upper class [27]. The cut-off for each expenditure group was updated using 2019 data from the Bureau of Statistics and was converted into household levels in our study questionnaire [28]. Smoking status indicated whether a person actively smoked cigarettes within the past month.

Second, variables related to protective behaviors included knowledge of PPE standards, application of the six steps of handwashing, the use of PPE when in contact with suspected or positive COVID-19 patients, physical distancing, the use of a mask outside of the home, and the index of handwashing frequency. Regarding the knowledge of PPE standards, we asked whether the respondents knew about the minimum PPE requirement for their jobs at healthcare facilities based on the recommendation of the Ministry of Health 2020. We also asked whether a person always applies the six-step hand washing technique recommended by the WHO, maintains physical distancing, and uses a mask outside of the home. Additionally, a handwashing index was created as a proxy of handwashing behaviors, using a weighted factor analysis based on 4-point-Likert-scale questions, which asked whether respondents use hand sanitizer or wash their hands using soap on several essential occasions. These occasions included: (1) after being in a public place, (2) before eating, (3) after using the toilet, and (4) after touching animals or taking out trash. The designated occasions were developed based on the Center for Disease Control’s ten critical handwashing times [29]. In the analysis, we used a dummy variable indicating whether a person’s handwashing index was above or equal to the median value.

### 2.3. Data Analysis

We employed three statistical analyses: descriptive analysis, bivariate analysis, and multivariate logistic regressions. We conducted data analyses for hospital staff (healthcare and non-healthcare workers), healthcare workers, and non-healthcare workers. We conducted bivariate analyses to assess the correlation between each independent variable and COVID-19 infection, and we performed multivariate logistic regressions to assess the socioeconomic and behavioral correlates of infection. We reported odds ratios (ORs), adjusted odds ratios (AORs), confidence intervals, and *p*-values. All analyses were performed in STATA 15 and used a 5% level of statistical significance.

## 3. Results

Table 1 provides the sample characteristics. In sociodemographic terms, 82.6% of the sample were healthcare workers and 17.9% were non-healthcare workers, 62.2% were female, 77.6% were 25–44 years old, 54.5% had a 3–4 household size, 35.9% were poor or vulnerable, and 10.2% actively smoked. In terms of protective behavior (Table 1B), among all samples, 98.4% knew of PPE standards, 79.0% reported doing the six-step handwashing technique, 55% reported always using PPE when in contact with actual or suspected COVID-19 cases. Additionally, 61.7% had a high index of handwashing frequency, 41.7% reported always keeping physical distance, and 92.3% reported always using masks outside of the home. In terms of dependent variables, 1.57% of the samples had confirmed COVID-19. In terms of COVID-19 symptoms, 4.2%, 16.9%, 14.2%, and 1.7% of the samples had a fever, cough, sore throat, and shortness of breath, respectively.

By subgroup, the characteristics of healthcare workers and non-healthcare workers varied. Healthcare worker samples were primarily female (66%), and non-healthcare worker samples were mainly males (56%). Additionally, 79.8% vs. 67.1% of healthcare workers and non-healthcare workers were 25–44 years old, 33.7% vs. 46.1% of healthcare workers and non-healthcare workers were poor or vulnerable, and 5.8% vs. 31.3% of healthcare workers and non-healthcare workers were smokers. Furthermore, healthcare workers were shown to have higher infection rates, at 1.73%, than non-healthcare workers, at 0.82%. Healthcare workers reported higher rates of application of the six-step handwashing technique, knowledge of PPE standards, PPE usage when in contact with suspected/positive patients, and handwashing frequency.

Table 2 provides the bivariate (OR) and multivariate (AOR) analyses of all samples and healthcare workers. Note that the results for non-healthcare workers were not reported here because most independent variables were omitted in the regressions (potentially because the number of infections was very low). In the multivariable analysis, among all samples, higher risks of COVID-19 infection were significantly associated with the status of being healthcare workers (AOR = 8.31, 95% CI 1.27–54.54). In terms of socioeconomic correlates, the results show that the male sex, a larger household size, a higher expenditure level, and not smoking were associated with higher risks of infection. However, only a household size of more than five (AOR = 4.09, 95% CI 1.02–16.43) was statistically significant at a 5% level. In terms of protective behaviors, the results show that knowledge of PPE standards, always applying handwashing techniques, always using PPEs when in contact with suspects or cases, always applying physical distancing, and always using a mask outside of the home were associated with lower risks of infection. However, only knowledge of PPE standards (AOR = 0.08, 95% CI 0.01–0.54) and applying the six steps of handwashing (AOR = 0.32, 95% CI 0.12–0.83) were statistically significant at a 5% level.

Table 3 provides additional results for multivariate (AOR) analyses using at least one main symptom as the outcome variable. Among all samples, in terms of socioeconomic correlates, the results show that the female sex, a younger age group (19–24 years), a smaller household size, a higher expenditure level, and smoking were associated with a higher rate of at least one main symptom. However, only the expenditure level showed statistical significance. In terms of protective behaviors, knowledge of PPE standards, always applying handwashing techniques, using PPE when in contact with suspected or known cases, applying physical distancing, and using a mask outside of the home were associated with a lower rate of at least one main symptom. However, only always using PPE when in contact with suspected or known cases showed statistical significance.

## 4. Discussion

Our findings show that larger household sizes and middle to upper expenditure levels were significantly associated with higher risks of COVID-19 infection among hospital workers. Additionally, knowledge of PPE standards and use of PPE and frequency of application of the six-step handwashing technique were significant correlates of lower risks of infection. Our results also showed that sociodemographic variables (e.g., sex and age) and behavioral variables (e.g., physical distancing, the use of a mask, and the index of handwashing frequency) were associated with higher or lower risks of infection but were not statistically significant. This may be due to not having a large enough sample, given the very low infection rates in the sample (1.57%). Note that the results for all samples may be mainly driven by the characteristics of the healthcare workers.

The analysis of all samples revealed that being a healthcare worker was positively correlated with COVID-19 status. In other words, the infection rates were significantly higher among healthcare workers compared to non-healthcare workers, which was similar to a study in China, which showed that the infection rates were 2.10% and 0.43% among healthcare and non-healthcare workers, respectively [30]. The results also corroborate findings from previous studies, which discovered that the infection risk of healthcare workers was significantly higher than that of non-healthcare workers [8,22,30]. The positive association between being a healthcare worker and COVID-19 status may be explained by several factors experienced particularly by healthcare workers, such as performing certain medical procedures, prolonged contact with infected patients, and working pressures during the pandemic period [14,16,31].

We also found a significant association between larger household size and infection. This result is consistent with previous studies indicating positive relationships between household size and COVID-19 infection in the general population [32,33,34]. A possible link between the two indicators is that the within-household infection rate is higher than the non-household one, so that the larger household size may increase contacts and spread of SARS-CoV-2 [35]. In terms of expenditure levels, we found that being in the middle and upper expenditure levels was protective of contracting COVID-19, which supports evidence from previous studies that low socioeconomic status and expenditure may increase the risk of COVID-19 infection [17,36]. A potential explanation may be the lower compliance of lower-expenditure people in applying protective measures, such as wearing masks, physical distancing, and washing hands [37,38] and the lower immune system of those with a lower socioeconomic status due to higher stress levels and a higher allostatic load, which makes them more susceptible to COVID-19 [39,40,41,42].

In our study, knowledge of standard PPE and use of PPE when in contact with suspects or patients showed protective effects of COVID-19 infections among all samples and healthcare samples. However, the effect of the latter was only significant at the 10% level. Similarly, previous studies have shown that knowledge of the disease and proper use of PPE have an inverse association with being infected with SARS-CoV-1 [43], another coronavirus type that previously caused an epidemic. It has been suggested that the proper use of various types of PPE, adequate provision of PPE, and sufficient access to PPE may protect healthcare workers from contracting COVID-19 [14,18,19]. Although the negligible effect of the use of PPE in this study was unexpected, the direction of the correlation is still consistent with earlier studies.

To our knowledge, there is currently no study evaluating the effect of the six-step hand washing technique on COVID-19 status among healthcare workers. Our finding is supportive of other studies showing that handwashing frequency, especially in contact with patients, may protect healthcare workers from being infected by SARS-CoV-2 and SARS-CoV-1 [21,39,44,45]. The significant correlation of the indicator may also stem from the hypothesis that applying the six-step hand washing technique is biologically more effective than implementing non-six-step handwashing techniques [46].

Our study had several limitations. First, we used self-administered questionnaires for sample characteristics and behaviors. This may pose risks of under- or over-reporting. Second, this was a cross-sectional study, which may be improved in future investigations by applying cohort studies to draw statistical inferences. Despite the limitations, this study provides further evidence that hospital workers face challenges in combating COVID-19 at work. Besides the higher infection risk of the healthcare workers, as found in the current study, previous research also discovered overwhelming workload burdens of healthcare workers that may lead to some health and psychological problems such as greater sleep disorders and headache episodes [47] and more depressive, anxiety, and burnout symptoms [48]. To ensure that healthcare and non-healthcare workers, particularly those in LMICs, can make significant contributions to combat the pandemic and indirectly generate potential economic impacts for the country [49,50], further efforts are needed to provide adequate knowledge and training of proper PPE use and to supply sufficient standardized PPE in contact with patients.

## 5. Conclusions

Our study assessed the socioeconomic and behavioral correlates of COVID-19 infections among healthcare workers at eight hospitals in the Greater Jakarta Area, the capital of Indonesia. We found that healthcare workers were at significantly higher risks of contracting COVID-19 compared to non-healthcare workers at hospitals. We also found that socioeconomic correlates such as a larger household size and middle and upper expenditure levels were significantly associated with higher risks of infection. Moreover, protective behaviors such as knowledge and use of PPE and frequency of applying the six-step handwashing technique were significantly associated with lower risks among hospital workers. These findings add to the evidence of the determinants of COVID-19 infections of healthcare and non-healthcare workers at hospitals in LMICs.

## Figures and Tables

**Table 1:** Sample characteristics.

Variables	(1)		(2)		(3)	
	Hospital Workers (*N* = 1397)		Healthcare Workers (*N* = 1154)		Non-Healthcare Workers (*N* = 243)	
	*n*	%	*n*	%	*n*	%
(A) Demographics						
Sex						
Female	869	62.2	762	66.03	107	44.03
Male	528	37.8	392	33.97	136	55.97
Age group						
19–24 years	126	9.020	83	7.190	43	17.700
25–44 years	1084	77.59	921	79.81	163	67.08
>44 years	187	13.39	150	13	37	15.23
Household size						
1–2	268	19.18	229	19.84	39	16.05
3–4	761	54.47	636	55.11	125	51.44
≥5	368	26.34	289	25.04	79	32.51
Expenditure class						
Poor	202	14.46	163	14.12	39	16.05
Vulnerable	299	21.4	226	19.58	73	30.04
Aspiring middle class	600	42.95	502	43.5	98	40.33
Middle and upper class	296	21.19	263	22.79	33	13.58
Active smoking status						
No	1254	89.76	1087	94.19	167	68.72
Yes	143	10.24	67	5.81	76	31.28
(B) Protective behavior						
Knowledge of PPE standards						
No	22	1.570	8	0.690	14	5.760
Yes	1375	98.43	1146	99.31	229	94.24
Application of the six-step hand washing technique						
Otherwise	294	21.050	238	20.620	56	23.050
Always	1103	78.95	916	79.38	187	76.95
The use of PPEs when in contact with suspected/positive COVID-19 patients						
Otherwise	627	44.880	479	41.510	148	60.910
Always	770	55.12	675	58.49	95	39.09
Index of hand-washing frequency						
Low	535	38.300	441	38.210	94	38.680
High	862	61.7	713	61.79	149	61.32
Physical distancing						
Otherwise	814	58.27	698	60.49	116	47.74
Always	583	41.73	456	39.51	127	60.49
The use of a mask outside of the home						
Otherwise	108	7.73	91	7.89	17	7
Always	1289	92.27	1063	92.11	226	93
(C) Signs and symptoms						
Fever	58	4.15	47	4.07	11	4.53
Cough	236	16.89	197	17.07	39	16.05
Runny nose	198	14.17	175	15.16	23	9.47
Sore throat	198	14.17	175	15.16	23	9.47
Shortness of breath	24	1.72	18	1.56	6	2.47
Common cold	58	4.15	51	4.42	7	2.88
Headache	171	12.24	139	12.05	32	13.17
Muscle ache	129	9.23	109	9.45	20	8.23
Nausea	70	5.01	59	5.11	11	4.53
Watery eyes	22	1.57	20	1.73	2	0.82
Sputum production	125	8.95	102	8.84	23	9.47
Dizziness	79	5.65	61	5.29	18	7.41
Rash on skin	20	1.43	18	1.56	2	0.82
Loss of appetite	41	2.93	33	2.86	8	3.29
Anosmia	12	0.86	11	0.95	1	0.41
Ageusia	12	0.86	11	0.95	1	0.41
Tingling sensation	26	1.86	20	1.73	6	2.47
Delirium	6	0.43	1	0.09	5	2.06
(D) Dependent variables						
RT-PCR result						
Negative	1375	98.43	1134	98.27	241	99.18
Positive	22	1.57	20	1.73	2	0.82
Having at least one main symptom						
No	1124	80.46	923	79.98	201	82.72
Yes	273	19.54	231	20.02	42	17.28
Data are *n*/*N* (%) if not specified						

**Table 2:** Unadjusted and adjusted odds ratios of factors associated with COVID-19 infection.

Variables	(1)		(2)		(3)		(4)	
	Healthcare Workers		Healthcare Workers		Hospital Workers		Hospital Workers	
	(*N* = 1154)		(*N* = 1007)		(*N* = 1397)		(*N* = 1397)	
	OR (CI 95%)	*p*-Value	AOR (CI 95%)	*p*-Value	OR (CI 95%)	*p*-Value	AOR (CI 95%)	*p*-Value
(A) Demographics								
Sex								
Female	Ref		Ref		Ref		Ref	
Male	1.05 (0.41–2.65)	0.922	1.90 (0.68–5.29)	0.222	1.14 (0.48–2.69)	0.762	1.91 (0.71–5.16)	0.201
Age group								
19–24 years	Ref		Ref		Ref		Ref	
25–44 years	0.58 (0.13–2.62)	0.478	0.75 (0.16–3.63)	0.723	0.54 (0.15–1.89)	0.333	0.66 (0.19–2.32)	0.513
>44 years	1.40 (0.26–7.37)	0.694	2.31 (0.40–13.38)	0.351	1.13 (0.26–4.80)	0.872	2.16 (0.50–9.35)	0.301
Status of being a healthcare worker								
No					Ref		Ref	
Yes	NA	NA	NA	NA	2.13 (0.49–9.16)	0.312	8.31 (1.27–54.54)	0.027
Household size								
1–2	Ref		Ref		Ref		Ref	
3–4	2.00 (0.44–9.09)	0.371	2.94 (0.76–11.42)	0.12	2.13 (0.47–9.59)	0.324	3.03 (0.75–12.15)	0.118
≥5	2.82 (0.58–13.70)	0.199	3.69 (0.92–14.84)	0.066	2.96 (0.62–14.04)	0.173	4.09 (1.02–16.43)	0.047
Expenditure class								
Poor	Ref		Ref		Ref		Ref	
Vulnerable	1.21 (0.28–5.13)	0.799	0.79 (0.17–3.70)	0.768	0.67 (0.19–2.35)	0.531	0.50 (0.14–1.76)	0.282
Aspiring middle class	1.19 (0.33–4.34)	0.787	0.68 (0.16–2.99)	0.613	0.74 (0.25–2.14)	0.574	0.44 (0.13–1.45)	0.175
Middle and upper class	0.20 (0.02–1.98)	0.17	0.084 (0.01–1.21)	0.069	0.13 (0.02–1.15)	0.067	0.06 (0.01–0.66)	0.022
Active smoking status								
No					Ref		Ref	
Yes	NA	NA	NA	NA	0.41 (0.06–3.10)	0.39	0.43 (0.07–2.58)	0.355
(B) Protective behavior								
Knowledge of PPE standards								
No	Ref		Ref		Ref		Ref	
Yes	0.12 (0.01–1.01)	0.051	0.06 (0.00–0.63)	0.02	0.15 (0.03–0.67)	0.014	0.08 (0.01–0.54)	0.01
Application of the six-step hand washing technique								
Otherwise	Ref		Ref		Ref		Ref	
Always	0.48 (0.19–1.20)	0.117	0.30 (0.11–0.83)	0.02	0.46 (0.19–1.11)	0.083	0.32 (0.12–0.83)	0.019
The use of PPEs when in contact with suspected/positive COVID-19 patients								
Otherwise	Ref		Ref		Ref		Ref	
Always	0.47 (0.19–1.15)	0.098	0.38 (0.13–1.09)	0.073	0.46 (0.19–1.10)	0.082	0.37 (0.13–1.02)	0.055
Index of hand-washing frequency								
Low	Ref		Ref		Ref		Ref	
High	0.75 (0.31–1.83)	0.53	0.75 (0.26–2.12)	0.587	0.62 (0.27–1.43)	0.26	0.61 (0.23–1.60)	0.317
Physical distancing								
Otherwise	Ref		Ref		Ref		Ref	
Always	1.54 (0.64–3.74)	0.337	2.42 (0.81–7.22)	0.114	1.40 (0.60–3.26)	0.43	2.52 (0.6–7.42)	0.092
The use of a mask outside of the home								
Otherwise					Ref		Ref	
Always	NA	NA	NA	NA	1.77 (0.24–13.31)	0.578	3.44 (0.42–27.99)	0.248

Note: OR = odds ratio; AOR = adjusted odds ratio; Ref = reference group; NA = not applicable. We also performed bivariate and multivariable analyses among non-healthcare workers, but most independent variables were omitted potentially because the number of COVID-19 infections was very low.

**Table 3:** Unadjusted and adjusted odds ratios of factors associated with experiencing at least one of COVID-19’s main symptoms.

Variables	(1)		(2)		(3)	
	Healthcare Workers		Non-Healthcare Workers		All Samples	
	*N* = 1154		*N* = 243		(*N* = 1397)	
	AOR (CI 95%)	*p*-Value	AOR (CI 95%)	*p*-Value	AOR (CI 95%)	*p*-Value
(A) Demographics						
Sex						
Female	Ref		Ref		Ref	
Male	0.84 (0.60–1.19)	0.329	1.01 (0.43–2.37)	0.974	0.84 (0.61–1.14)	0.26
Age group						
19–24 years	Ref		Ref		Ref	
25–44 years	0.58 (0.33–1.00)	0.051	1.58 (0.40–5.01)	0.438	0.73 (0.45–1.19)	0.213
>44 years	0.68 (0.34–1.35)	0.267	2.03 (0.51–8.10)	0.313	0.87 (0.47–1.62)	0.671
Status of being a healthcare worker						
No					Ref	
Yes	NA	NA	NA	NA	1.36 (0.89–2.08)	0.153
Household size						
1–2	Ref		Ref		Ref	
3–4	0.91 (0.62–1.34)	0.637	0.57 (0.23–1.40)	0.219	0.84 (0.59–1.19)	0.332
≥5	0.79 (0.50–1.25)	0.316	0.78 (0.27–2.29)	0.656	0.78 (0.52–1.17)	0.232
Expenditure class						
Poor	Ref		Ref		Ref	
Vulnerable	1.38 (0.81–2.37)	0.239	2.48 (0.69–9.96)	0.201	1.46 (0.90–2.36)	0.127
Aspiring middle class	1.56 (0.95–2.55)	0.076	2.94 (0.71–12.16)	0.136	1.66 (1.06–2.59)	0.027
Middle and upper class	1.13 (0.64–2.00)	0.664	2.16 (0.42–11.06)	0.353	1.20 (0.71–2.02)	0.489
Active smoking status						
No	Ref		Ref		Ref	
Yes	1.40 (0.73–2.65)	0.31	0.78 (0.28–2.16)	0.63	1.13 (0.66–1.93)	0.658
(B) Protective behavior						
Knowledge of PPE standards						
No	Ref		Ref		Ref	
Yes	0.27 (0.07–1.07)	0.063	1.35 (0.24–7.72)	0.735	0.63 (0.24–1.66)	0.348
Application of WHO hand-washing steps						
Otherwise	Ref		Ref		Ref	
Always	0.85 (0.58–1.23)	0.386	0.63 (0.30–1.33)	0.224	0.82 (0.59–1.15)	0.258
The use of PPE when in contact with suspected/positive COVID-19 patients						
Otherwise	Ref		Ref		Ref	
Always	0.61 (0.45–0.83)	0.002	0.64 (0.30–1.38)	0.254	0.63 (0.47–0.83)	0.001
Index of hand-washing frequency						
Low	Ref		Ref		Ref	
High	0.73 (0.53–1.01)	0.06	1.60 (0.71–3.61)	0.254	0.81 (0.6–1.10)	0.178
Physical distancing						
Otherwise	Ref		Ref		Ref	
Always	1.00 (0.71–1.42)	0.993	0.64 (0.29–1.40)	0.264	0.93 (0.68–1.27)	0.646
The use of a mask outside of the home						
Otherwise	Ref		Ref		Ref	
Always	0.68 (0.41–1.14)	0.142	0.76 (0.22–2.70)	0.676	0.67 (0.42–1.07)	0.095

Note: dependent variable = dummy, having at least one main symptom; OR = odds ratio; AOR = adjusted odds ratio; ref = reference group; NA = not applicable.

## Data Availability

Available upon reasonable request.
